# Literary Notices

**Published:** 1878-08-20

**Authors:** 


### LITERARY NOTICES.

Berberidaceæ. — The botanical description, commercial history, medical properties and pharmaceutical preparations. By C. G. & J. N. Lloyd, Cincinnati.

This is an interesting pamphlet of 16 pages, which the author proposes to furnish to any member of the profession who will enclose a stamp to cover postage.

The native genera of the nat. ord. Berberidaceæ are Berberis, Caulophyllumthalictroides, Dyphyllum, Jeffersonia Podophyllum, Vancouveria and Achyls. These several varieties are described by the author, and a beautiful illustration of the Berberis is given.

The Sunny South—A weekly paper, devoted to Literature, Romance, Science, Education, Temperance and Southern progress. J. H. & W. B. Seals editors and proprietors, Mrs. Mary E. Bryan associate editor, Atlanta, Ga. Terms, $3 per annum in advance.

We take pleasure in recommending the above paper to our readers as an excellent one. The Messrs. Seals are both able and experienced journalists, and have brought to their aid the editorial services of Mrs. Mary E. Bryan, of whose great talent and popularity as a writer the Southern people are justly proud.

The ruinous and suicidal habit of the Southern people to disparage and neglect all literary enterprises in their midst, can find no shadow of pretext in the case of the Sunny South, as it is fully equal, if not superior to the best Northern journals, and is eminently worthy of patronage and support.

W.

Cholecystotomy, for the removal of gall-stones in dropsy of the gall-bladder, by J. Marion Sims, M.D., founder of the Woman's Hospital of the State of New York, ex-President of American Medical Association, etc.. etc.

An interesting paper giving an account of the removal of gall-stones from the gall-bladder by a remarkable operation, which, though skilfully performed, resulted unfavorably.

The best Pocket Anatomist—By C. Henry Leonard, A.M. M.D., Detroit, Michigan, pp. 60. Price 50 cents.

A very useful little work for medical students.

Antagonism of Alcohol and Diphtheria, by E. N. Chapman, A. M., M.D., Prof. of Materia Medica and Therapeutics and Clinical Midwifery, etc., etc.

A neat little work of 98 pages, containing both theoretical and practical suggestions which should be examined by the profession.

MEDICAL ELECTRICITY.—Electro vapor and medicated baths for the cure of chronic and specific diseases not amenable to ordinary remedies. By Dr. C. V. Meador, Little Rock, Ark.

A neat little work of 30 pages.

ATLAS OF SKIN DISEASES, by Louis A. Duhring, M. D., Prof. of Skin Diseases in the Hospital of the University of Pennsylvania etc. Part IV. Philadelphia, J. B. Lippincott & Co., 1878. Price $2.50 per part.

On a former occasion we referred to this excellent work. In the present number we have life illustrations of vitiligo, alopecia, areata, tinea favosa, eczema rubrum. Every medical man ought to possess himself of this beautiful, practical and useful work of Prof. Duhring.

